# Dr. T. Clifford Allbutt

**Published:** 1870-09

**Authors:** 


Dr. T. Clifford Allbutt writes to the *Lancet* in earnest advocacy of the open-air treatment of disease, more especially as regards the management of typhus. He states, that while in charge of the Fever Hospital, at Leeds, his experience led him to increase ventilation, until at last the windows were nailed open, so that during a winter epidemic of typhus, "the nurses had to wear bonnets, or other head-coverings, and the breezes played freely about the beds." The mortality was greatly lessened, and there were no pulmonary or other internal complications. Dr. Allbutt regards the open-air treatment as of more importance than that by cold water. "It probably acts somewhat similarly, and it is easy of management, while the cold water system presents almost insuperable difficulties in extensive hospital practice."—*Med. Gazette.*

